# On the Excretions as Guides to Treatment in Rheumatism

**Published:** 1854-07

**Authors:** 


### *On the Excretions as guides to treatment in Rheumatism.*

BY DR. FULLER.

The author began by stating that no great advance can take place in our own knowledge of dieease, nor any material improvement in its treatment, unless we endeavour to discover the primary cause of each morbid action, and trace its influence in modifying and deranging the various functions of life. After briefly illustrating this important truth, he proceeded to point out how close a relationship the amount and character of the various exertions must necessarily bear to the condition of the general system, and how certain an index they afford to the energy of those processes by which the effete materials of the body are got rid of. Hence he deduced the inference, that no plan of treatment can be proposed, with a well-founded rational prospect of success, which is not based on a due regard to the different excretions, and varied with their varying condition.

He then proceeded to apply this general law to the elucidation of the treatment of rheumatism and rheumatic gout, and showed that, inasmuch as these disorders depend on the presence of morbid matter, the product of imperfect or faulty assimilation, a proper action of the excretory organs is more than usually necessary. The alterations usually produced on the character of the exertions by the existence of rheumatism and rheumatic gout, were next alluded to, and some remarkable exceptions pointed out; and the author stated his opinion that the chief aim of treatment should be, by producing, as far as possible, an incnease of those exeretions which are scanty or deficient, to make each and all of the excretery organs assist in eliminating the *materies morbi*, and to endeavour, by close attention to the character of the excretions, to correct their morbid condition. He then referred to the good effects resulting from treatment regulated according to these views, and mentioned many facts to prove and illustrate the ill success which attends every mode of treatment in which the condition of the excretory organs is not attended to. Having fully established these general principles, his next endeavor was to point out the means by which they can best be carried out. He first premised that if all the excretions are scanty or suppressed, and if at the same time the pulse be full and bounding, venesection will not only relieve the general tension of the system, and alleviate the pain and general distress, but will be followed by action of the excretory organs. He then proceeded to discuss each of the excretions separately, and in regard to the prespiration, stated his conviction that much mischief is often done by interfering with Nature's mode of operation. No bath should be administered as long as prespiration takes place naturally, but if the skin is dry or acting sluggishly, a bath is essential to stimulate its action. He strongly recommended a water bath of 100° Fahr., rendered alkaline by potash or soda, but in the event of its being impracticable to make use of a water bath, the vapour or hot-air bath may be substituted. In either case the effects of the bath should be sustained by guaiacum and Dover's powder, or tartarized antimony and saline diaphoretic medicines. The only exceptions to this general rule are met with in persons of weakly constitution, or towards the close of lingering cases. In such instances the prespiration is sometimes very profuse, but loses its distinctive emphyrheumatic odour, and much of its peculiar acid character, and is accompanied by a soddened state of the skin, a quick, feeble, irritable pulse, and not unfrequently by an eruption of sudamina. Tonics, such as quina and sulphuric acid, are their requsite, instead of diaphoretics and salines, and as soon as all feverishness has subsided, the cautious admiuistration of iron is almost always beneficial. The urine was next appealed to, and made to furnish its quota of evidence. Dr. Fuller insisted strongly on the fact that the mere appearence of the urine, its color, clearness, or torpidity, affords no clue to its real condition—to the amount and character of its solid ingredients, which can only be ascertained by careful examination. This he proved by reference to facts, and then went on to show that the amount of solid matter excreted by the kidneys is usually much diminished, and that diuretics are necessary to increase their action. A most important question is, as to what diuretics should be employed. A state of congestion and irritation exists consequent on the abnormal condition of the blood, and the exhibition of ordinary diuretic medicines, which operate merely as renal stimulants, is more likely to increase that congestion, than to cause an abundant flow of urine. Hence cantharides, squills, nitric ether, whilst alkalies and the neutral salts, such as the acetate of potash and the potassio-tartrate of soda, which correct the condition of the blood, are most active in promoting diuresis. So also are the preparations of colchicum. Water, too, proves of service by promoting the absorption of the salts, and assisting not only in the excretion of the solid matters, but in their subsequent solution. The condition of the urine, as the specific gravity, turbidity, and activity, was shown to the best practical test as to the dose in which alkalies should be administered, the frequency of their repetition, and the property of persevering in their use. The alvine evacuations were next referred to, the necessity for strict attention to their character was pointed out, and the peculiar conditions which call for the administration of different remedies were clearly indicated. Dr. Fuller insisted upon the powerful cholagogue influence of aloes and the acetous extract of colchicum in these cases, and blue pill or calomel, whenever it appears desirable to excite an increased flow of bile.—The principles of treatment already laid down were next applied to chronic rheumatism, and subsequently to rheumatic gout, and it was shown that in the latter form of disease the treatment requistie to produce the desired effects need considerable modification according to the stage of the disorder, and the constitution of the patient. A disregard of this fact, together with the practice, too prevalent in the present day, of prescribing each medicine separately, constitute, in Dr. Fuller's opinion, the chief cause of the frequent failure of the treatment ordinarily employed in rheumatism and rheumatic gout, and form additional grounds for a close examination of the excreta, inasmuch as such an examination proves that no two cases are alike, but necessarily require remedies differing widely in their character, no less than in the dose in which, and the period of the attack at which they should be administered.

Dr. Semple, after speaking of the unsatisfactory results of the treatment of rheumatism which he had formerly pursued, by bleeding &c., observed, that for some time past he had treated all cases of acute rheumatism with lemon-juice, and the result had been invariably satisfactory. For the first few days of treatment he placed the patient on strictly low diet, and administered the juice of six lemons daily; this, with opium, given in the form of a soap-pill, formed his entire treatment. The opium was given in doses sufficient to relieve the pain, and might consist of one, two, or even three grains. Under this plan the pain gradually became less, the fever subsided, and the disease abated. The return to health was more rapid than when exhausting treatment had been resorted to. Care was requisite, after convalescence, that the diet was not too stimulating. This treatment had the advantage of allowing us to use more energetic measures when any local complication, as heart disease, took place, as the system had not been previously exhausted by treatment. The plan had also the advantage of simplicity, and surely that was a very great one.

He did not profess to determine the *modus operandi* of the lemon-juice, but its beneficial results were unmistakable.

Dr. Theophilus Thompson thought that in Dr. Fuller's paper the importance of the state of the excretions as an indication of the mode of treatment to be employed, had been overrated. The author had accidentally touched upon a more important point—viz: the state of the blood in rheumatism. In this disease we must have a carefui regard to the general condition of the patient, as well as paying a strict attention to the state of secretions, for a similar state of the excretions might exist under very different conditions of the system, and different modes of treatment be therefore indicated. The excretions, too, instead of being guides to treatment, might only show that the disease was passing off. Respecting lemon-juice in rheumatism, he had found it of more service in inflammatory cases of the disease, in which the patient was not robust, and deleption could not be resorted to.—*London Lancet.*

